# Prognostic impact of white blood cell counts on clinical outcomes in patients with chronic renal insufficiency undergoing percutaneous coronary intervention

**DOI:** 10.3389/fcvm.2023.1027107

**Published:** 2023-03-09

**Authors:** Wei Yan, Mengyao Li, Yumeng Lei, Shuaiyong Zhang, Fengfeng Lv, Jiawang Wang, Qian Yang, Na Yu, Ming Chen, Xufen Cao, Liqiu Yan

**Affiliations:** ^1^Department of Cardiology and Dongguan Cardiovascular Research Institute, Dongguan Songshan Lake Central Hospital, Guangdong Medical University, Dongguan, China; ^2^Department of Cardiology, Cangzhou Central Hospital, Hebei Medical University, Cangzhou, China

**Keywords:** white blood cell count, SYNTAX score, SYNTAX score II, chronic renal insufficiency, percutaneous coronary intervention

## Abstract

**Objective:**

To determine whether the inclusion of white blood cell (WBC) counts in the SYNTAX score (SS) or SS II models could improve the models’ performance for risk stratification in individuals with chronic renal insufficiency (CRI) following percutaneous coronary intervention (PCI).

**Methods:**

In total, 2,313 patients with CRI, who were subjected to PCI and had data available on in-hospital WBC (ih-WBC) counts, were recruited. Patients were divided into 3 groups as per their ih-WBC counts (low, medium, and high). The primary endpoints were all-cause mortality (ACM) and cardiac mortality (CM). The secondary endpoints incorporated myocardial infarction, stroke, unplanned revascularization, and major adverse cardiovascular and cerebrovascular events (MACCEs).

**Results:**

During a median follow-up of 3 years, the high WBC group had the highest incidences of CM (2.4% vs. 2.1% vs. 6.7%; *p* < 0.001), ACM (6.3% vs. 4.1% vs. 8.2%; *p* < 0.001), unplanned revascularization (8.4% vs. 12.4% vs. 14.1%; *p* < 0.001), and MACCEs (19.3% vs. 23.0% vs. 29.2%; *p* < 0.001) among the three groups. Multivariable Cox regression analysis depicted that the risk of ACM and CM in the high WBC group was 2.577 (95% confidence interval [CI]: 1.504–4.415, *p* < 0.001) and 3.850 (95% CI: 1.835–8.080, *p* < 0.001) times that in the low WBC group after adjusting for other confounding factors. A combination of ih-WBC counts with SS or SS II significantly improved the risk assessment and prediction of ACM and CM.

**Conclusion:**

The ih-WBC counts was associated with the risk of occurrence of ACM, CM, unplanned revascularization, and MACCEs in individuals with CRI following PCI. It provides an incremental predictive value for the occurrence of ACM and CM when included in SS or SS II models.

## 1. Introduction

Inflammation exerts a critical function in the progression as well as plaque destabilization in atherosclerosis (1). Studies have found that downstream biomarkers of inflammation, such as interleukin-6 and the high-sensitivity C-reactive protein (hs-CRP) are linked to a greater risk of cardiovascular events (2, 3).

The white blood cell (WBC) counts are a widely-used and easily available marker of inflammation in clinical practice. Its predictive value as a marker for mortality in individuals with acute coronary syndrome (ACS) is well-established (4–6). Recently, it has also been demonstrated that total, as well as differential in-hospital white blood cell (ih-WBC) counts, are independent prognostic factors for long-term deaths and major adverse cardiovascular and cerebrovascular events (MACCEs). Including ih-WBC counts in SYNTAX score (SS) or SYNTAX score II (SS-II) models can improve mortality predictions in individuals with triple-vessel coronary artery disease (CAD) (7). However, there have been no assessments of the predictive value of ih-WBC counts in patients with CRI after percutaneous coronary intervention (PCI). Chronic renal insufficiency (CRI), which is defined as the presence of kidney damage or reduced kidney function (estimated glomerular filtration rate (eGFR) <90 mL/min/1.73 m^2^) for ≥3 months (8), is high-risk comorbidity that increases the risk of cardiovascular mortality and morbidity and is known to be associated with poorer clinical outcomes in patients following PCI (9). In this investigation, we sought to evaluate the utility of including ih-WBC counts as a factor in SS or SS II models for anticipating long-term clinical outcomes in individuals with CRI following PCI.

## 2. Methods

### 2.1. Study population

**Figure 1:**
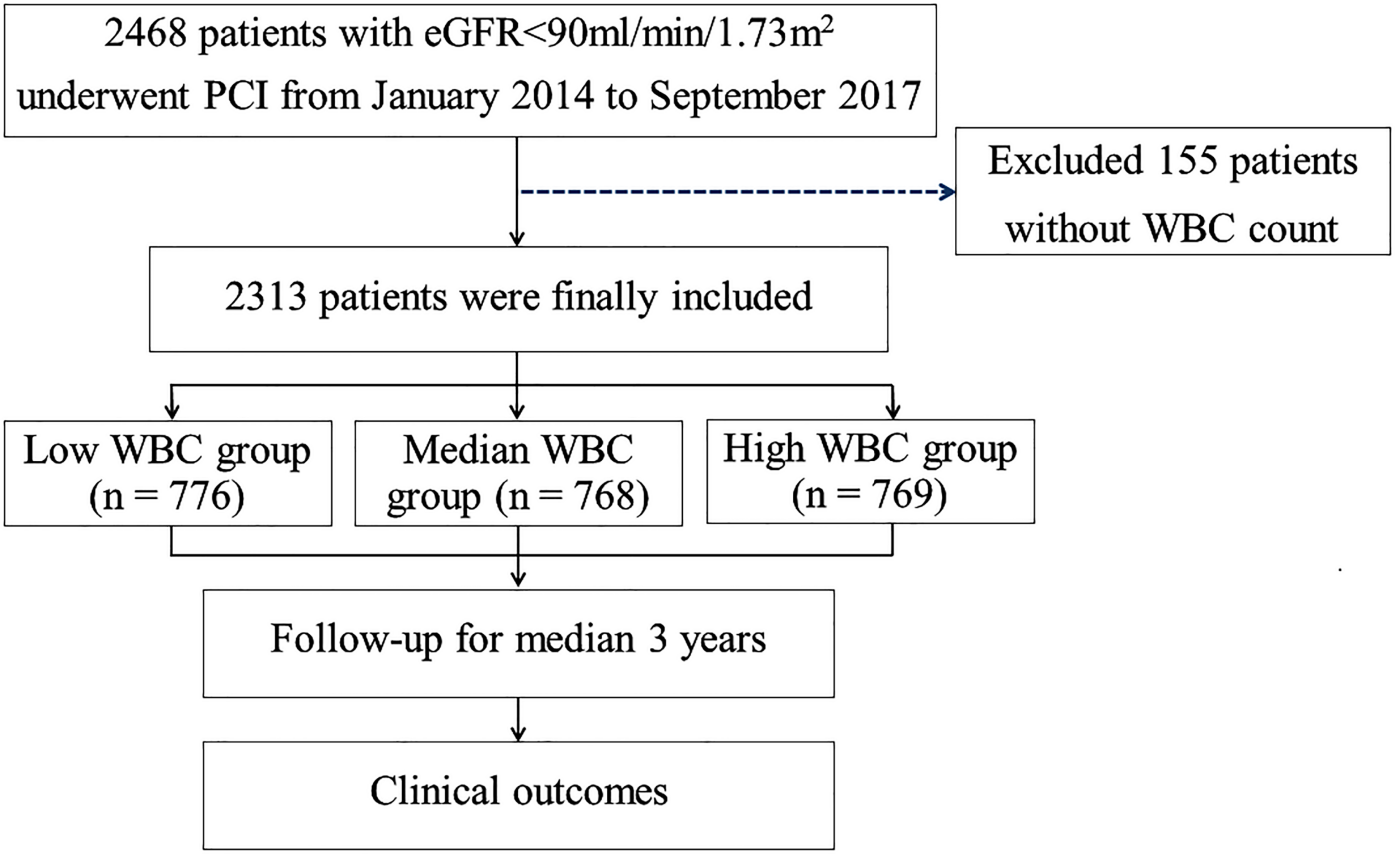
Study flow chart. PCI, percutaneous coronary intervention; eGFR, estimated glomerular filtration rate; CABG, coronary artery bypass graft; WBC, white blood cell.

The study design of the risk evaluation of CRI patients following PCI has been described previously (10). Briefly, a total of 2,468 patients with creatinine clearance rates (CrCl) <90 mL/min per 1.73 m^2^ who underwent PCI between January 2014 and September 2017 in Cangzhou Central Hospital, Hebei Medical University, were retrospectively enrolled in the study. After excluding 155 patients for whom ih-WBC counts data were not available, 2,313 patients were included in this investigation. Patients were classified into three groups as per tertiles of ih-WBC counts as follows: low WBC group [WBC counts ≤6.18*10^9^/L (*n* = 776)], medium WBC group [WBC counts >6.18*10^9^/L but ≤8.14*10^9^/L (*n* = 768)], and high WBC group [WBC counts >8.14*10^9^/L (*n* = 769)] (Figure 1). The study protocol was subjected to approval by the ethics committee of Cangzhou Central Hospital. Written and informed consent was obtained for all subjects.

The clinical and interventional data of the participants were collected from the electronic medical record (EMR) system. The CrCl values were calculated utilizing the simplified Modification of Diet in Renal Disease (MDRD) equation. The ih-WBC counts were defined as the first WBC counts value from the EMR system. Two of three trained cardiologists (who were blinded to the clinical data as well as outcomes) calculated the SS (11) and SS II (12) through the dedicated website.^1^ In case of any disagreement, the opinion of a third observer was obtained and resolved by consensus.

### 2.2. Study endpoints and follow-up

Clinical follow-up was performed *via* clinic visits or telephone conversations. The primary endpoints included all-cause mortality (ACM) as well as cardiac mortality (CM). Deaths that could not be attributed to non-cardiac causes were considered CM. The secondary endpoints included myocardial infarction (MI), stroke, unplanned revascularization, and major adverse cardiovascular and cerebrovascular events (MACCEs), defined as a composite of ACM, MI, stroke, and unplanned revascularization. MI was defined following the consensus document on the fourth universal definition (13). Stroke was defined as neural dysfunction due to a sudden rupture or blockage of a blood vessel, and was diagnosed based on signs of brain dysfunction or imaging evidence (14). Revascularization of PCI or coronary artery bypass grafting (CABG) driven by ischemic symptoms or cardiac events was defined as unplanned revascularization. All endpoints were confirmed by two independent clinicians.

### 2.3. Statistical analysis

Continuous variables are reported as mean ± standard deviation (SD) or medians with interquartile ranges (IQR). Categorical variables are presented as frequencies and percentages. Continuous variables were compared utilizing one-way ANOVAs or Kruskal-Wallis tests, when necessary. Chi-square or the Fisher exact tests, on the other hand, carried out comparisons of categorical variables. Patients who were lost to follow-up were deemed at risk until they were censored at the date of the last contact. The cumulative event rates were measured utilizing Kaplan–Meier curves and compared across groups *via* the log-rank test. We assessed the prognostic value of ih-WBC grouping for predicting clinical outcomes using multivariable Cox regression models. While Log[−logS(t)] plots were used to test for proportional hazard assumption. All potential confounders (with *p* < 0.1 in the univariate analyses) were incorporated in the multivariate analyses. By combining ih-WBC counts with SS or SS II values, we assessed the improvements in model performance, risk classification, and discrimination; this was done by comparing the AUC of the two nested models employing the nonparametric deLong approach and computing the net reclassification improvement (NRI) as well as the integrated discrimination improvement (IDI) indices. A two-sided value of *p* < 0.05 was statistically significant. SPSS 24.0 (IBM Corp., Armonk, NY, United States) and R Software Version 3.6.0 (The R Foundation for Statistical Computing, Vienna, Austria) conducted all the statistical analyses of this investigation.

## 3. Results

### 3.1. Patients’ baseline characteristics

The ih-WBC counts ranged from 2.6*10^9^/L to 32.7*10^9^/L. The SS values ranged from 1.0 to 47.0, and the SS-II values ranged from 9.7 to 59.6. Patients with high ih-WBC counts were younger and more likely to have current smoker status; clinical presentation of MI; reduced eGFR; lower left ventricular ejection fraction (LVEF); higher left ventricular end-diastolic diameter (LVEDD); and elevated levels of serum creatinine, blood glucose, total cholesterol, triglyceride, and low-density lipoprotein (*p* < 0.05 or *p* < 0.001) than those with low and median ih-WBC counts. Patients with high ih-WBC counts also had higher baseline SS values and were more likely to have thrombus lesions and undergo primary PCI (*p* < 0.001 for all) (Tables 1, 2).

**Table 1:** Baseline clinical characteristics of patients.

	Low ih-WBC count (*n* = 776)	Medium ih-WBC count (*n* = 768)	High ih-WBC count (*n* = 769)	*P*-value
*Demographics*				
Age, yrs	66.0 (61.0–72.0)	66.0 (60.0–71.0)	65.0 (59.0–70.0)	<0.001
Male	409 (52.7)	471 (61.3)	468 (60.9)	0.001
BMI, kg/m^2^	25.7 ± 3.4	26.4 ± 3.3	25.8 ± 3.2	0.012
*Previous history*				
Diabetes	148 (19.1)	190 (24.7)	189 (24.6)	0.010
Hypertension	521 (67.1)	550 (71.6)	484 (62.9)	0.001
Dyslipidemia	266 (34.3)	341 (44.4)	322 (41.9)	<0.001
Current smoker	61 (7.9)	90 (11.7)	110 (14.3)	<0.001
Prior MI	69 (8.9)	69 (9.0)	58 (7.5)	0.524
Previous PCI	101 (13.0)	116 (15.1)	89 (11.6)	0.121
Previous stroke	82 (10.6)	94 (12.2)	76 (9.9)	0.312
COPD, n (%)	11 (1.4)	12 (1.6)	14 (1.8)	0.815
*Clinical presentation*				<0.001
Stable angina	428 (55.2)	352 (45.8)	172 (22.4)	
Unstable angina	132 (17.0)	113 (14.7)	56 (7.3)	
NSTEMI	103 (13.3)	143 (18.6)	158 (20.5)	
STEMI	113 (14.6)	160 (20.8)	383 (49.8)	
eGFR, ml/min	79.9 (71.9–85.7)	78.5(69.8–85.0)	77.8(67.3–84.6)	<0.001
LVEF, %	63.0 (59.0–66.0)	62.0 (58.0–66.0)	60.0 (53.0–65.0)	<0.001
LVEDD (mm)	47.0 (45.0–50.0)	47.0 (44.0–51.0)	48.0 (45.0–53.0)	0.006
*Baseline laboratory*				
WBC, *10^9^/L	5.3 (4.8–5.8)	7.1 (6.6–7.6)	10.1 (8.9–11.9)	<0.001
Hemoglobin (mg/dL)	13.1 (12.0–14.0)	13.4 (12.4–14.4)	13.2 (11.9–14.4)	<0.001
Creatinine (mg/dL)	0.93 (0.78–1.02)	0.96 (0.84–1.07)	0.97 (0.85–1.1)	<0.001
Blood glucose (mg/dL)	107.5 (93.6–139.5)	111.6 (95.4–147.7)	132.8 (107.6–187.2)	<0.001
Total cholesterol (mg/dL)	164.4 (143.1–193.4)	170.2 (143.1–196.9)	174.0 (150.0–205.1)	<0.001
TG (mg/dL)	124.0 (93.0–174.5)	139.1 (98.3–195.1)	140.4 (103.6–200.2)	<0.001
HDL (mg/dL)	35.6 (30.2–41.4)	35.2 (29.8–41.0)	36.0 (30.9–41.4)	0.275
LDL (mg/dL)	94.0 (76.2–114.8)	96.3 (77.2–120.4)	102.1 (84.6–123.1)	<0.001

Values are mean ± SD, median (IQR), or n (%). BMI, body mass index; MI, myocardial infarction; PCI, percutaneous coronary intervention; COPD, chronic obstructive pulmonary disease; NSTEMI, non-ST-segment elevation myocardial infarction; STEMI, ST-segment elevation myocardial infarction; eGFR, estimated glomerular filtration rate; LVEF, left ventricular ejection fraction; LVEDD, left ventricular end diastolic diameter; TG, triglyceride; HDL, high density lipoprotein; LDL, low density lipoprotein.

**Table 2:** Anatomical characteristics of lesions and procedural details.

	Low ih-WBC count (*n* = 776)	Medium ih-WBC count (*n* = 768)	High ih-WBC count (*n* = 769)	*P*-value
*CAD extension*				0.070
1-vessel disease	187 (24.1)	161 (21.0)	157 (20.4)	
2-vessel disease	265 (34.1)	282 (36.7)	247 (32.1)	
3-vessel disease	324 (41.8)	325 (42.3)	365 (47.5)	
Left main disease	49 (6.3)	50 (6.5)	47 (6.1)	0.950
*Lesion characteristics*				
Lesion length > 20 mm	445 (57.3)	429 (55.9)	362 (47.1)	<0.001
Bifurcation or trifurcation	241 (31.1)	198 (25.8)	153 (19.9)	<0.001
Aorto-ostial lesion	13 (1.7)	15 (2.0)	24 (3.1)	0.127
Heavy calcification	86 (11.1)	68 (8.9)	49 (6.4)	0.005
Severe tortuosity	46 (5.9)	50 (6.5)	40 (5.2)	0.550
Thrombus	24 (3.1)	40 (5.2)	226 (29.4)	<0.001
Chronic total occlusions	97 (12.5)	133 (17.3)	101 (13.1)	0.014
Target vessel number	1 (1–1)	1 (1–2)	1 (1–1)	0.099
*Target lesion location*				
LM	27 (3.5)	24 (3.1)	17 (2.2)	0.314
LAD	444 (57.2)	404 (52.6)	391 (50.8)	0.035
LCX	212 (27.3)	247 (32.2)	195 (25.4)	0.010
RCA	307 (39.6)	324 (42.4)	352 (45.8)	0.046
*Procedural characteristics*				
Stent per patient	2.0 (1.0–2.0)	2.0 (1.0–2.0)	1.0 (1.0–2.0)	0.016
Total length of stent, mm	41.0 (25.0–64.0)	38.0 (24.0–64.0)	36.0 (25.0–60.0)	0.151
Stent length > 100 mm	65 (8.4)	48 (6.3)	43 (5.6)	0.074
Mean stent diameter, mm	3.00 (2.75–3.25)	2.95 (2.75–3.25)	3.00 (2.75–3.25)	0.683
Minimum stent diameter, mm	2.75 (2.50–3.00)	2.75 (2.50–4.00)	2.75 (2.50–3.00)	0.266
Maximum stent diameter, mm	3.00 (2.75–3.50)	3.00 (2.75–3.50)	3.00 (2.75–3.50)	0.669
Primary PCI	13 (1.7)	25 (3.3)	191 (24.8)	<0.001
Baseline SYNTAX score	8.0 (13.0–18.5)	8.0 (13.0–18.5)	14.0 (9.0–19.5)	0.004
SYNTAX II score for PCI	28.3 (24.3–32.3)	27.6 (23.6–32.1)	28.2 (23.5–33.0)	0.165

Values are mean ± SD, median (IQR), or n (%). CAD, coronary artery disease; LM, left main; LAD, left anterior descending artery; LCX, left circumflex; RCA, right coronary artery.

### 3.2. Association of ih-WBC counts with clinical outcomes

**Figure 2:**
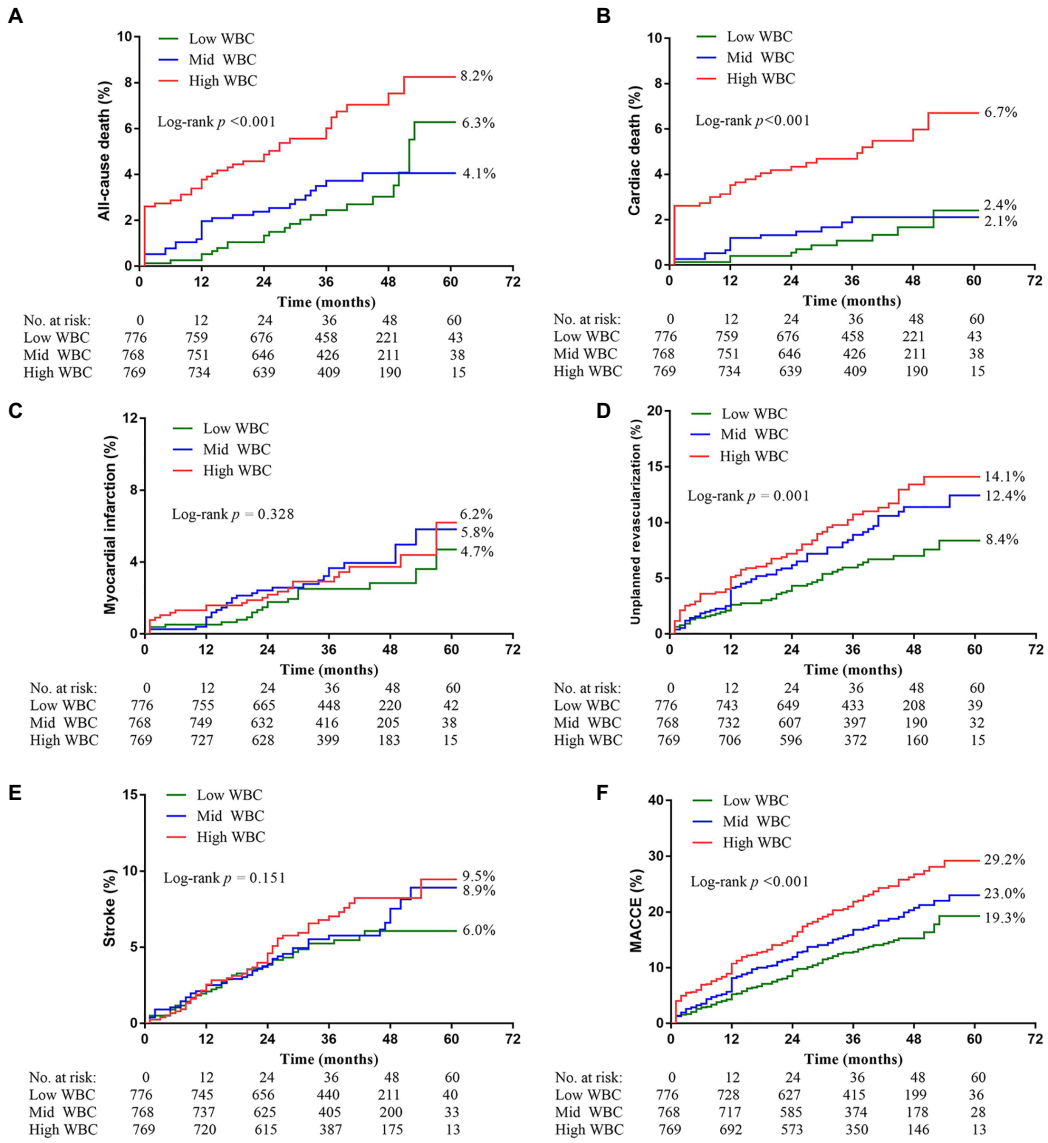
Kaplan–Meier curves showing event rates stratified by the WBC counts. **(A)** All-cause death. **(B)** Cardiac death. **(C)** Myocardial infarction. **(D)** Unplanned revascularization. **(E)** Stroke. **(F)** Major adverse cardiovascular and cerebrovascular events (MACCE).

The median follow-up period was 3 years (IQR = 1.5–5.0). Among the three groups, the high ih-WBC counts group had the highest 5-year cumulative event rates of ACM (6.3% vs. 4.1% vs. 8.2%; *p* < 0.001), CM (2.4% vs. 2.1% vs. 6.7%; p < 0.001), unplanned revascularization (8.4% vs. 12.4% vs. 14.1%; *p* < 0.001), and MACCEs (19.3% vs. 23.0% vs. 29.2%; *p* < 0.001) (Table 3 and Figure 2). Univariate Cox regression analyses for different clinical outcomes were shown in Supplementary Table 1. Multivariate Cox regression analyses affirmed that the risk of ACM and CM in the high WBC group was 2.577 (95% confidence interval [CI]: 1.504–4.415, *p* < 0.001) and 3.850 (95% CI: 1.835–8.080, p < 0.001) times that in the low WBC group after adjusting for other confounding factors (Table 4).

**Table 3:** Five-year cumulative incidence of adverse events.

	Low ih-WBC count (a)	Medium ih-WBC count (b)	High ih-WBC count (c)	*P*-value			
				Trend	a vs. b*	b vs. c*	a vs. c*
All-cause mortality	6.3% (49)	4.1% (55)	8.2% (63)	<0.001	0.599	0.006	0.001
Cardiac mortality	2.4% (19)	2.1% (16)	6.7% (52)	<0.001	0.377	<0.001	<0.001
Myocardial infarction	4.7% (36)	5.8% (45)	6.2% (48)	0.328	0.197	0.778	0.313
Unplanned revascularization	8.4% (65)	12.4% (95)	14.1% (108)	0.001	0.024	0.275	0.001
Stroke	6.0% (47)	8.9% (68)	9.5% (73)	0.151	0.510	0.435	0.154
MACCEs	19.3 (150)	23.0% (177)	29.2% (225)	<0.001	0.042	0.008	<0.001

Event rates are Kaplan–Meier estimates, %(n). *Adjusted significance level is 0.017. MACCEs, major adverse cardiovascular and cerebrovascular events.

**Table 4:** Multivariate Cox regression analyses for median 3-year clinical outcomes.

Variables	All-cause mortality		Variables	Cardiac mortality	
	Hazard ratio (95% CI)	*P*-value		Hazard ratio (95% CI)	*P*-value
WBC_Low	Ref.		WBC_Low	Ref.	
WBC_Mid	1.293(0.731–2.289)	0.377	WBC_Mid	1.427(0.627–3.247)	0.396
WBC_High	2.577(1.504–4.415)	<0.001	WBC_High	3.850(1.835–8.080)	<0.001
Age	1.097(1.066–1.130)	<0.001	Age	1.079(1.040–1.119)	<0.001
Hypertension	0.667(0.442–1.006)	0.053	Diabetes	1.115(0.622–2.001)	0.715
Dyslipidemia	0.631(0.367–1.085)	0.096	Dyslipidemia	0.617(0.350–1.089)	0.096
COPD	3.395(1.614–7.144)	0.001	COPD	3.953(1.667–9.374)	0.002
Stable angina	Ref.		Stable angina	Ref.	
Unstable angina	2.184(1.372–3.476)	0.491	Unstable angina	1.105(0.657–1.858)	0.708
NSTEMI	0.967(0.576–1.623)	0.598	NSTEMI	1.150(0.605–2.185)	0.670
STEMI	1.200(0.671–2.143)	0.983	STEMI	0.760(0.353–1.637)	0.483
eGFR	0.972(0.948–0.996)	0.023	eGFR	0.966(0.937–0.995)	0.024
LVEF	0.971(0.946–0.998)	0.038	LVEF	0.986(0.950–1.023)	0.445
LVEDD	1.006(0.966–1.048)	0.762	LVEDD	1.032(0.971–1.098)	0.304
Hemoglobin	0.999(0.988–1.012)	0.992	Hemoglobin	0.999(0.984–1.014)	0.878
Creatinine	0.994(0.982–1.006)	0.307	Creatinine	0.992(0.978–1.006)	0.276
Blood glucose	1.030(0.988–1.074)	0.161	Blood glucose	1.035(0.991–1.082)	0.122
TG	0.943(0.716–1.242)	0.675	SYNTAX score	1.024(0.995–1.055)	0.111
SYNTAX score	1.012(0.987–1.037)	0.363			
Variables	Unplanned revascularization		Variables	Stroke	
	Hazard ratio (95% CI)	*P*-value		Hazard ratio (95% CI)	*P*-value
WBC_Low	Ref.		Age	1.034(1.012–1.056)	0.002
WBC_Mid	1.184(0.772–1.818)	0.439	Male	0.585(0.390–0.878)	0.010
WBC_High	1.389(0.915–2.109)	0.123	Hypertension	1.435(0.963–2.140)	0.076
BMI	1.035(0.958–1.118)	0.386	Previous stroke	1.963(1.296–2.975)	0.001
Dyslipidemia	0.846(0.569–1.258)	0.409	eGFR	1.002(0.986–1.018)	0.801
Prior MI	1.132(0.635–2.017)	0.675	Creatinine	1.008(1.004–1.012)	<0.001
Previous PCI	1.145(0.695–1.887)	0.595	Total cholesterol	0.932(0.630–1.379)	0.724
Total cholesterol	0.862(0.570–1.304)	0.483	LDL	0.954(0.569–1.600)	0.859
LDL	0.961(0.567–1.630)	0.883	SYNTAX score	1.019(0.998–1.041)	0.080
SYNTAX score	1.025(1.004–1.047)	0.022			
Variables	Myocardial infarction		Variables	MACCEs	
	Hazard ratio (95% CI)	*P*-value		Hazard ratio (95% CI)	*P*-value
BMI	1.058(0.939–1.192)	0.354	WBC_Low	Ref.	
Diabetes	1.134(0.648–1.984)	0.660	WBC_Mid	1.184(0.772–1.818)	0.552
Prior MI	1.290 (0.532–3.129)	0.049	WBC_High	1.389(0.915–2.109)	0.217
Previous PCI	1.846 (0.943–3.612)	0.010	Age	1.036(1.014–1.059)	0.001
Stable angina	Ref.		Male	0.526(0.347–0.796)	0.002
Unstable angina	2.184(1.372–3.476)	<0.001	BMI	1.029(0.950–1.114)	0.489
NSTEMI	0.967(0.576–1.623)	0.899	Diabetes	1.128(0.721–1.763)	0.598
STEMI	1.200(0.671–2.143)	0.539	Prior MI	1.207(0.675–2.159)	0.525
eGFR	0.991(0.970–1.012)	0.400	Previous PCI	1.062(0.642–1.756)	0.815
LVEDD	1.007(0.955–1.062)	0.792	COPD	1.467(0.462–4.662)	0.516
Creatinine	1.000(0.993–1.008)	0.929	Stable angina	Ref.	
Blood glucose	1.018(0.965–1.074)	0.514	Unstable angina	1.134(0.801–1.604)	0.478
TG	1.108(0.940–1.306)	0.220	NSTEMI	1.086(0.745–1.583)	0.667
SYNTAX score	1.025(0.995–1.055)	0.102	STEMI	1.268(0.823–1.952)	0.282
			eGFR	0.996(0.980–1.012)	0.607
			LVEF	1.036(1.004–1.069)	0.025
			LVEDD	0.993(0.949–1.040)	0.776
			Creatinine	1.006(1.003–1.011)	<0.001
			Blood glucose	1.004(0.958–1.053)	0.860
			SYNTAX score	1.020(0.999–1.043)	0.065

CI, confidence interval; WBC, white blood cell; BMI, body mass index; MI, myocardial infarction; PCI, percutaneous coronary intervention; COPD, chronic obstructive pulmonary disease; eGFR, estimated glomerular filtration rate; LVEF, left ventricular ejection fraction; LVEDD, left ventricular end diastolic diameter; TG, triglyceride; LDL, low density lipoprotein; MACCEs, major adverse cardiovascular and cerebrovascular events; NSTEMI, non-ST-segment elevation myocardial infarction; STEMI, ST-segment elevation myocardial infarction.

### 3.3. Combination of ih-WBC counts with SS or SS II values for prediction of ACM and CM

**Figure 3:**
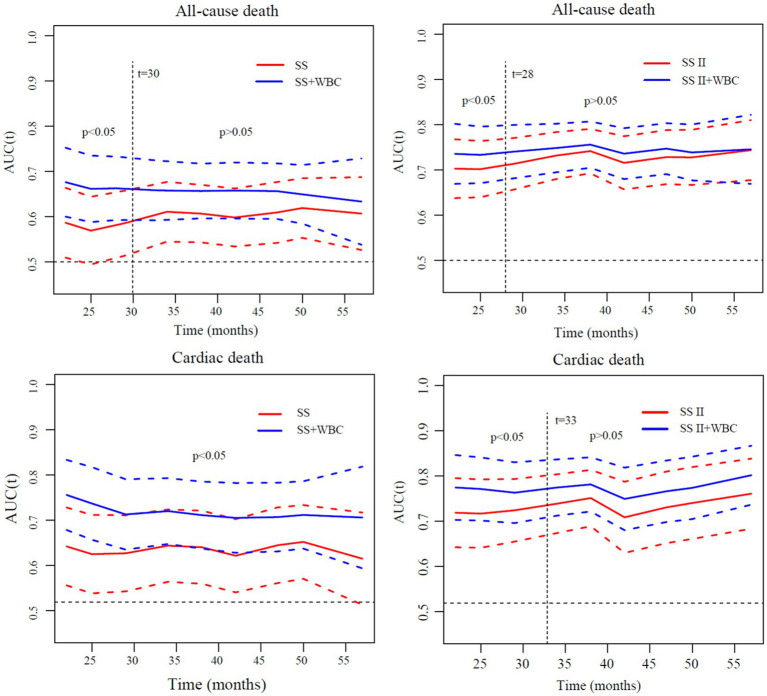
Comparisons of time-dependent AUCs of different models for discrimination of all-cause death and cardiac death. AUC, area under curve; SS, SYNTAX score; SS II, SYNTAX score II; WBC, white blood cell; SS II+WBC: SYNTAX score II plus white blood cell.

The analyses of time-dependent AUCs for ACM showed that the AUCs for SS in combination with ih-WBC counts were significantly larger than those of SS alone (*p* < 0.05) during the 30-month follow-up period. However, the degree of increase tended to decrease with time. There was no remarkable difference between the predictive value of the SS plus ih-WBC counts model and the SS model (*p* > 0.05) when the follow-up times were longer than 30 months. A similar result was observed for the SS-II models, albeit with a cutoff value of 28 months instead of 30 months. In the models used to predict the incidence of CM, though the extent of increase also tended to decrease with time, there was a remarkable difference between the predictive value of the SS plus ih-WBC counts model and the SS model (*p* < 0.05) during the whole follow-up period, and for the SS-II models, the cutoff value was 33 months (Figure 3).

Furthermore, by combining the ih-WBC counts with SS or SS II models, the metrics for risk reclassification and discrimination were significantly improved. The respective NRIs of the SS plus ih-WBC counts model over the SS model were 0.121 for ACM and 0.188 for CM; the NRIs of the SS-II plus ih-WBC counts model over the SS-II model were 0.025 for ACM and 0.135 for CM. The IDI indices of the SS plus ih-WBC counts model over the SS model were 0.019 (*p* < 0.01) for ACM and 0.022 (*p* < 0.001) for CM and the IDI indices of the SS-II plus ih-WBC counts model over the SS-II model were 0.025 (*p* < 0.001) for ACM and 0.032 (*p* < 0.001) for CM (Table 5).

**Table 5:** Statistics for model improvement of for all-cause mortality and cardiac mortality.

	Discrimination		Risk reclassification				
	IDI [95% CI]	*P*-value	Events		Non-events		NRI [95% CI]
			Risk up	Risk down	Risk up	Risk down	
*All-cause mortality*							
SS vs. SS + WBC	0.019 (0.004–0.058)	<0.01	0.243	0.092	0.113	0.083	0.121 (−0.003–0.232)
SS II vs. SS II + WBC	0.025 (0.005–0.060)	<0.001	0.119	0.106	0.056	0.069	0.025 (−0.051–0.239)
Cardiac mortality							
SS vs. SS + WBC	0.022 (0.006–0.060)	<0.001	0.256	0.033	0.089	0.045	0.188 (0.023–0.322)
SS II vs. SS II + WBC	0.032 (0.009–0.060)	<0.001	0.209	0.077	0.060	0.057	0.135 (−0.051–0.266)

CI, confidence interval; AUC, area under the curve; SS, SYNTAX score; SS II, SYNTAX score II; WBC, white blood cell; NRI, net reclassification improvement; IDI, integrated discrimination improvement.

## 4. Discussion

This study shows that the ih-WBC counts is associated with the risk of occurrence of ACM, CM, unplanned revascularization, and MACCEs in individuals with CRI following PCI. The ih-WBC counts can be utilized for risk reclassification, especially in secondary prevention among patients with CRI post-PCI. Integrating ih-WBC counts into SS or SS II models also improves the predictive performance of these models and facilitates the identification of patients at risk for future ACM and CM. Therefore, the ih-WBC counts may also be used to flag patients at risk for adverse cardiac events post-PCI, who may warrant more intensive follow-up and preventive treatment.

Inflammation is known to exert an important function not only in atherogenesis but also in atherosclerotic plaque rupture resulting in acute coronary syndrome (ACS) (15–18). Studies have found that biomarkers of inflammation including hs-CRP and interleukin-6 are independent risk factors for cardiovascular events (19). Preprocedural hs-CRP elevation has been linked to a greater risk of adverse cardiac events in people undergoing PCI (20–22). The WBC counts, which can be easily and repeatedly obtained in clinical practice, are one of the most viable inflammatory biomarkers. Cannon et al. (4) have reported that WBC counts are linked to an increased rate of mortality after 30 days and 10 months in individuals with acute MI and unstable angina pectoris. The TACTICS-TIMI 18 [Treat Angina with Aggrastat and Determine Cost of Therapy with an Invasive or Conservative Strategy (TACTICS) Thrombolysis in Myocardial Infarction (TIMI)] sub-study demonstrated that an increased WBC counts predicted extensive CAD and increased mortality at 6 months in people with ACS (5). More recently, Alkhalfan et al. (23) observed that elevated WBC counts were linked to increased major or minor hemorrhage and ischemic events (such as cardiovascular death, MI, and stroke) in patients with ACS.

The SS model is a well-established tool used to predict adverse clinical outcomes to help clinicians decide on optimum revascularization strategies in individuals with complex CAD (11, 24, 25). The SS-II model incorporates the anatomical variables in the SS model with other clinical variables (age, sex, LVEF, CrCl, chronic obstructive pulmonary disease, and peripheral vascular disease), and can predict 4-year mortality with higher accuracy. The SS-II model is also a better guide than the SS model for decisions on PCI and CABG in complex CAD cases (12). Subsequently, several studies have demonstrated the predictive value of the SS-II model for predicting outcomes in different cohorts such as three-vessel and/or unprotected left main coronary artery disease (ULMAD) following PCI (26, 27), ACS (28, 29), and cardiogenic shocks after primary PCI (30).

The SS-II model was created according to a Cox proportional hazards model utilizing the SYNTAX trial findings (12). The baseline features that were strongly associated with 4-year mortality were added to the model. However, the ih-WBC counts, a marker of the inflammatory state, were not included in the SS-II model. A recent study observed that a combination of differential WBC (eosinophil, monocyte, and lymphocyte) counts enhanced the success of risk prediction and reclassification for mortality when combined with SS or SS II models in patients with triple-vessel CAD (7). Patients with CRI have a hallmark feature of persistent, low-grade inflammation, which is involved in the development of ACM (31). Inflammation plays an important role in the initiation and progression of kidney disease. Recent studies have reported that plasma proinflammatory biomarkers, such as soluble TNF receptors 1 and 2 (TNFR-1 and TNFR2) were associated with the increased risk of progression of diabetic kidney disease, even after adjustment for established clinical risk factors (32, 33). However, not much is known about the predictive value of ih-WBC counts for predicting clinical outcomes in patients with CRI post-PCI. This study demonstrated that elevated ih-WBC counts were associated with increased incidence of ACM, CM, unplanned revascularization, and MACCEs in individuals with CRI following PCI. Furthermore, the addition of ih-WBC counts to the SS or SS II models also improved the predictive performance of these models in predicting ACM and CM events in individuals with CRI following PCI, although the degree of improvement in predictive performance tended to decrease with time.

## 5. Limitations

Despite these promising results, our results should be viewed in the light of multiple limitations. First, this study is based on data from a single center and is retrospective and observational in nature; therefore, it can only identify associations and cannot ascribe causality to related events. Second, differences in blood collection periods from the occurrence of the index event were not controlled for in this analysis. Third, we did not collect information on differential WBC counts and hs-CRP levels, both of which may be important for clinical outcomes. Finally, in some patients, we believe that the ih-WBC counts data may have been affected by undetected infections or other conditions for which we have no information.

## 6. Conclusion

In patients with CRI following PCI, an elevated ih-WBC counts was found to be associated with the risk of occurrence of ACM, CM, unplanned revascularization, and MACCEs. A combination of ih-WBC counts with SS or SS II models significantly improved these models’ performance in predicting ACM and CM.

## Data availability statement

The raw data supporting the conclusions of this article will be made available by the authors, without undue reservation.

## Ethics statement

The studies involving human participants were reviewed and approved by the Ethics Committee of Cangzhou Central Hospital, Hebei Medical University. The patients/participants provided their written informed consent to participate in this study.

## Author contributions

WY, LY, and ML provided the conception of the idea for the study and analyzed the acquired data. LY, WY, ML, SZ, and YL contributed to the development of the methodology and wrote the manuscript. FL, JW, NY, and MC were responsible for the interpretation of statistical results. XC revised the manuscript. All authors contributed to the article and approved the submitted version.

## Funding

This work was supported by the Natural Science Foundation of Hebei Province, China (H2021110008) and Hebei Provence Key Research Projects (172777163).

## Conflict of interest

The authors declare that the research was conducted in the absence of any commercial or financial relationships that could be construed as a potential conflict of interest.

## Publisher’s note

All claims expressed in this article are solely those of the authors and do not necessarily represent those of their affiliated organizations, or those of the publisher, the editors and the reviewers. Any product that may be evaluated in this article, or claim that may be made by its manufacturer, is not guaranteed or endorsed by the publisher.

## Footnotes

^1^ http://www.syntaxscore.com

## References

[1] Pedro-Botet J Climent E Benaiges D. Atherosclerosis and inflammation. New Ther Appr Med Clin. (2020) 155:256–62. doi: 10.1016/j.medcli.2020.04.024 32571617

[2] Ziegler L Gajulapuri A Frumento P Bonomi A Wallén H de Faire U. Interleukin 6 trans-signalling and risk of future cardiovascular events. Cardiovasc Res. (2019) 115:213–21. doi: 10.1093/cvr/cvy191, PMID: 30052808

[3] Shimizu T Suwa S Dohi T Wada H Miyauchi K Shitara J. Clinical significance of high-sensitivity C-reactive protein in patients with preserved renal function following percutaneous coronary intervention. Int Heart J. (2019) 60:1037–42. doi: 10.1536/ihj.18-683, PMID: 31484863

[4] Cannon CP McCabe CH Wilcox RG Bentley JH Braunwald E. Association of white blood cell count with increased mortality in acute myocardial infarction and unstable angina pectoris. OPUS-TIMI 16 investigators. Am J Cardiol. (2001) 87:a10:636–9, A10. doi: 10.1016/s0002-9149(00)01444-2, PMID: 11230853

[5] Sabatine MS Morrow DA Cannon CP Murphy SA Demopoulos LA DiBattiste PM. Relationship between baseline white blood cell count and degree of coronary artery disease and mortality in patients with acute coronary syndromes: a TACTICS-TIMI 18 (treat angina with Aggrastat and determine cost of therapy with an invasive or conservative strategy- thrombolysis in myocardial infarction 18 trial) substudy. J Am Coll Cardiol. (2002) 40:1761–8. doi: 10.1016/s0735-1097(02)02484-1, PMID: 12446059

[6] Lindahl B Toss H Siegbahn A Venge P Wallentin L. Markers of myocardial damage and inflammation in relation to long-term mortality in unstable coronary artery disease. FRISC study group. Fragmin during instability in coronary artery disease. N Engl J Med. (2000) 343:1139–47. doi: 10.1056/nejm200010193431602, PMID: 11036119

[7] Zhao X Jiang L Xu L Tian J Xu Y Zhao Y. Predictive value of in-hospital white blood cell count in Chinese patients with triple-vessel coronary disease. Eur J Prev Cardiol. (2019) 26:872–82. doi: 10.1177/2047487319826398, PMID: 30861699

[8] National Kidney Foundation. K/DOQI clinical practice guidelines for chronic kidney disease: evaluation, classification, and stratification. Am J Kidney Dis. (2002) 39:S1–S266. 11904577

[9] Tsai TT Messenger JC Brennan JM Patel UD Dai D Piana RN. Safety and efficacy of drug-eluting stents in older patients with chronic kidney disease: a report from the linked CathPCI registry-CMS claims database. J Am Coll Cardiol. (2011) 58:1859–69. doi: 10.1016/j.jacc.2011.06.056, PMID: 22018296

[10] Yan L Li P Wang Y Han D Li S Zhang J. Impact of the residual SYNTAX score on clinical outcomes after percutaneous coronary intervention for patients with chronic renal insufficiency. Catheter Cardiovasc Interv. (2020) 95:606–15. doi: 10.1002/ccd.28652, PMID: 31868307 PMC7078880

[11] Serruys PW Morice MC Kappetein AP Colombo A Holmes DR Mack MJ. Percutaneous coronary intervention versus coronary-artery bypass grafting for severe coronary artery disease. N Engl J Med. (2009) 360:961–72. doi: 10.1056/NEJMoa0804626 19228612

[12] Farooq V van Klaveren D Steyerberg EW Meliga E Vergouwe Y Chieffo A. Anatomical and clinical characteristics to guide decision making between coronary artery bypass surgery and percutaneous coronary intervention for individual patients: development and validation of SYNTAX score II. Lancet. (2013) 381:639–50. doi: 10.1016/s0140-6736(13)60108-7, PMID: 23439103

[13] Thygesen K Alpert JS Jaffe AS Chaitman BR Bax JJ Morrow DA. Fourth universal definition of myocardial infarction (2018). Circulation. (2018) 138:e618–51. doi: 10.1161/CIR.0000000000000617 30571511

[14] Li J Xin Y Li J Zhou L Qiu H Shen A. Association of haemoglobin glycation index with outcomes in patients with acute coronary syndrome: results from an observational cohort study in China. Diabetol Metab Syndr. (2022) 14:162. doi: 10.1186/s13098-022-00926-6, PMID: 36316759 PMC9620631

[15] Ross R. Atherosclerosis—an inflammatory disease. N Engl J Med. (1999) 340:115–26. doi: 10.1056/NEJM199901143400207 9887164

[16] Wolf D Ley K. Immunity and inflammation in atherosclerosis. Circ Res. (2019) 124:315–27. doi: 10.1161/CIRCRESAHA.118.313591, PMID: 30653442 PMC6342482

[17] Warnatsch A Ioannou M Wang Q Papayannopoulos V. Neutrophil extracellular traps license macrophages for cytokine production in atherosclerosis. Science. (2015) 349:316–20. doi: 10.1126/science.aaa8064, PMID: 26185250 PMC4854322

[18] Zakynthinos E Pappa N. Inflammatory biomarkers in coronary artery disease. J Cardiol. (2009) 53:317–33. doi: 10.1016/j.jjcc.2008.12.007 19477372

[19] Ridker PM Hennekens CH Buring JE Rifai N. C-reactive protein and other markers of inflammation in the prediction of cardiovascular disease in women. N Engl J Med. (2000) 342:836–43. doi: 10.1056/NEJM200003233421202, PMID: 10733371

[20] Wada H Dohi T Miyauchi K Shitara J Endo H Doi S. Preprocedural high-sensitivity C-reactive protein predicts long-term outcome of percutaneous coronary intervention. Circ. Soc. (2016) 81:90–5. doi: 10.1253/circj.CJ-16-0790 27867158

[21] Sabatine MS Morrow DA Jablonski KA Rice MM Warnica JW Domanski MJ. Prognostic significance of the Centers for Disease Control/American Heart Association high-sensitivity C-reactive protein cut points for cardiovascular and other outcomes in patients with stable coronary artery disease. Circulation. (2007) 115:1528–36. doi: 10.1161/circulationaha.106.649939, PMID: 17372173

[22] Razzouk L Muntner P Bansilal S Kini AS Aneja A Mozes J. C-reactive protein predicts long-term mortality independently of low-density lipoprotein cholesterol in patients undergoing percutaneous coronary intervention. Am Heart J. (2009) 158:277–83. doi: 10.1016/j.ahj.2009.05.026, PMID: 19619706

[23] Alkhalfan F Nafee T Yee MK Chi G Kalayci A Plotnikov A. Relation of white blood cell count to bleeding and ischemic events in patients with acute coronary syndrome (from the ATLAS ACS 2-TIMI 51 trial). Am J Cardiol. (2020) 125:661–9. doi: 10.1016/j.amjcard.2019.12.007, PMID: 31898965

[24] Sianos G Morel MA Kappetein AP Morice MC Colombo A Dawkins K. The SYNTAX score: an angiographic tool grading the complexity of coronary artery disease. EuroIntervention. (2005) 1:219–27. 19758907

[25] Morice M-C Serruys PW Kappetein AP Feldman TE Ståhle E Colombo A. Outcomes in patients with de novo left main disease treated with either percutaneous coronary intervention using paclitaxel-eluting stents or coronary artery bypass graft treatment in the synergy between percutaneous coronary intervention with TAXUS and cardiac surgery (SYNTAX) trial. Circulation. (2010) 121:2645–53. doi: 10.1161/circulationaha.109.899211 20530001

[26] Song Y Gao Z Tang X Ma Y Jiang P Xu J. Usefulness of the SYNTAX score II to validate 2-year outcomes in patients with complex coronary artery disease undergoing percutaneous coronary intervention: a large single-center study. Catheter Cardiovasc Interv. (2018) 92:40–7. doi: 10.1002/ccd.27321, PMID: 28895284

[27] Xu B Généreux P Yang Y Leon MB Xu L Qiao S. Validation and comparison of the long-term prognostic capability of the SYNTAX score-II among 1,528 consecutive patients who underwent left main percutaneous coronary intervention. J Am Coll Cardiol Intv. (2014) 7:1128–37. doi: 10.1016/j.jcin.2014.05.018 25240551

[28] Obeid S Frangieh AH Räber L Yousif N Gilhofer T Yamaji K. Prognostic value of SYNTAX score II in patients with acute coronary syndromes referred for invasive management: a subanalysis from the SPUM and COMFORTABLE AMI cohorts. Cardiol Res Pract. (2018) 2018:1–11. doi: 10.1155/2018/9762176, PMID: 30356345 PMC6176297

[29] Salvatore A Boukhris M Giubilato S Tomasello SD Castaing M Giunta R. Usefulness of SYNTAX score II in complex percutaneous coronary interventions in the setting of acute coronary syndrome. J Saudi Heart Assoc. (2016) 28:63–72. doi: 10.1016/j.jsha.2015.07.003, PMID: 27053895 PMC4803775

[30] Hayıroğlu Mİ Keskin M Uzun AO Bozbeyoğlu E Yıldırımtürk Ö Kozan Ö. Predictive value of SYNTAX score II for clinical outcomes in cardiogenic shock underwent primary percutaneous coronary intervention; a pilot study. Int J Cardiovasc Imaging. (2018) 34:329–36. doi: 10.1007/s10554-017-1241-9, PMID: 28889354

[31] Mihai S Codrici E Popescu ID Enciu A-M Albulescu L Necula LG. Inflammation-related mechanisms in chronic kidney disease prediction, progression, and outcome. J Immunol Res. (2018) 2018:1–16. doi: 10.1155/2018/2180373, PMID: 30271792 PMC6146775

[32] Schrauben SJ Shou H Zhang X Anderson AH Bonventre JV Chen J. Association of multiple plasma biomarker concentrations with progression of prevalent diabetic kidney disease: findings from the chronic renal insufficiency cohort (CRIC) study. J Am Soc Nephrol. (2021) 32:115–26. doi: 10.1681/ASN.2020040487, PMID: 33122288 PMC7894671

[33] Pavkov ME Weil EJ Fufaa GD Nelson RG Lemley KV Knowler WC. Tumor necrosis factor receptors 1 and 2 are associated with early glomerular lesions in type 2 diabetes. Kidney Int. (2016) 89:226–34. doi: 10.1016/j.kint.2016.06.002, PMID: 26398493 PMC4805514

