# Supplementary material for: Prognostic impact of white blood cell counts on clinical outcomes in patients with chronic renal insufficiency undergoing percutaneous coronary intervention
Source: Front Cardiovasc Med. 2023 Mar 9;10:1027107. doi: 10.3389/fcvm.2023.1027107 (PMC10034344; doi:10.3389/fcvm.2023.1027107)
Supplement: Supplementary file 1 [file Table_1.DOCX]

| **Supplementary Table 1. Univariate Cox regression analyses for clinical outcomes** | | | | | | | |
| --- | --- | --- | --- | --- | --- | --- | --- |
| **Variables** | **All-cause mortality** | | **Cardiac mortality** | | **Myocardial infarction** | |  |
|  | **HR (95% CI)** | **P-value** | **HR (95% CI)** | **P-value** | **HR (95% CI)** | **P-value** |  |
| Demographics |  |  |  |  |  |  |  |
| Age | 1.110(1.080-1.140) | <0.001 | 1.090(1.050-1.120) | <0.001 | 1.020(0.995-1.050) | 0.102 |  |
| Male | 1.080(0.723-1.610) | 0.712 | 1.010(0.615-1.660) | 0.971 | 0.961(0.603-1.530) | 0.867 |  |
| BMI | 0.937(0.860-1.020) | 0.138 | 0.979(0.879-1.090) | 0.696 | 1.090(0.990-1.200) | 0.079 |  |
| Previous history |  |  |  |  |  |  |  |
| Diabetes | 1.070(0.670-1.700) | 0.782 | 1.570(0.927-2.670) | 0.093 | 1.530(0.925-2.520) | 0.098 |  |
| Hypertension | 0.653(0.438-0.974) | 0.037 | 0.761(0.461-1.260) | 0.287 | 1.140(0.690-1.880) | 0.613 |  |
| Dyslipidemia | 0.515(0.329-0.807) | 0.004 | 0.563(0.327-0.971) | 0.039 | 1.050(0.659-1.670) | 0.844 |  |
| Current smoker | 1.030(0.550-1.930) | 0.925 | 1.330(0.655-2.680) | 0.432 | 0.601(0.242-1.490) | 0.272 |  |
| Prior MI | 1.380(0.735-2.580) | 0.318 | 1.780(0.880-3.600) | 0.108 | 2.410(1.320-4.380) | 0.004 |  |
| Previous stroke | 0.896(0.466-1.720) | 0.743 | 0.673(0.270-1.680) | 0.396 | 0.971(0.466-2.020) | 0.937 |  |
| Previous PCI | 1.020(0.569-1.830) | 0.945 | 1.230(0.627-2.420) | 0.546 | 2.070(1.200-3.560) | 0.009 |  |
| COPD | 6.370(3.090-13.100) | <0.001 | 7.200(3.100-16.700) | <0.001 | - | - |  |
| Clinical presentation |  |  |  |  |  |  |  |
| Stable angina | Ref. |  | Ref. |  | Ref. |  |  |
| Unstable angina | 1.520(1.060-2.180) | 0.023 | 2.220(1.390-3.540) | 0.001 | 2.180(1.410-3.370) | <0.001 |  |
| NSTEMI | 1.000(0.644-1.560) | 0.998 | 1.360(0.723-2.560) | 0.340 | 0.888(0.533-1.480) | 0.649 |  |
| STEMI | 0.940(0.565-1.560) | 0.812 | 0.666(0.310-1.430) | 0.296 | 1.140(0.638-2.020) | 0.667 |  |
| eGFR, ml/min | 0.970(0.959-0.981) | <0.001 | 0.963(0.951-0.976) | <0.001 | 0.983(0.968-0.998) | 0.024 |  |
| LVEF | 0.957(0.938-0.975) | <0.001 | 0.953(0.932-0.974) | <0.001 | 0.983(0.954-1.010) | 0.255 |  |
| LVEDD | 1.040(0.995-1.080) | 0.085 | 1.080(1.030-1.130) | 0.002 | 1.050(1.000-1.110) | 0.039 |  |
| Baseline laboratory |  |  |  |  |  |  |  |
| WBC_group | 1.550(1.200-1.990) | 0.001 | 2.220(1.570-3.130) | <0.001 | 1.150(0.868-1.530) | 0.328 |  |
| WBC_Low | Ref. |  | Ref. |  | Ref. |  |  |
| WBC_Mid | 1.160(0.662-2.030) | 0.604 | 1.430(0.636-3.220) | 0.386 | 1.460(0.820-2.580) | 0.199 |  |
| WBC_High | 2.250(1.370-3.700) | 0.001 | 4.200(2.100-8.400) | <0.001 | 1.350(0.749-2.430) | 0.320 |  |
| Hemoglobin | 0.983(0.972-0.994) | 0.002 | 0.980(0.966-0.993) | 0.003 | 0.990(0.977-1.000) | 0.158 |  |
| Creatinine | 1.000(1.000-1.010) | 0.014 | 1.000(1.000-1.010) | 0.004 | 1.000(1.000-1.010) | 0.046 |  |
| Blood glucose | 1.060(1.030-1.100) | 0.001 | 1.080(1.050-1.120) | <0.001 | 1.060(1.010-1.100) | 0.008 |  |
| Total cholesterol (mg/dl) | 0.938(0.766-1.150) | 0.532 | 0.933(0.727-1.200) | 0.589 | 1.090(0.874-1.360) | 0.447 |  |
| TG | 0.805(0.633-1.020) | 0.076 | 0.867(0.662-1.140) | 0.303 | 1.120(0.980-1.290) | 0.096 |  |
| HDL | 2.130(0.862-5.260) | 0.101 | 1.400(0.441-4.470) | 0.565 | 0.528(0.164-1.700) | 0.285 |  |
| LDL | 0.989(0.762-1.280) | 0.936 | 1.020(0.737-1.400) | 0.927 | 1.050(0.779-1.410) | 0.762 |  |
| SYNTAX score | 1.030(1.010-1.060) | 0.008 | 1.050(1.020-1.080) | 0.002 | 1.030(1.000-1.060) | 0.028 |  |
| **Variables** | **Stroke** | | **Unplanned revascularization** | | **MACCEs** | |  |
|  | **HR (95% CI)** | **P-value** | **HR (95% CI)** | **P-value** | **HR (95% CI)** | **P-value** |  |
| Demographics |  |  |  |  |  |  |  |
| Age | 1.030(1.010-1.050) | 0.002 | 0.997(0.981-1.010) | 0.703 | 1.030(1.020-1.040) | <0.001 |  |
| Male | 0.504(0.346-0.736) | <0.001 | 0.823(0.616-1.100) | 0.188 | 0.774(0.633-0.948) | 0.013 |  |
| BMI | 1.030(0.958-1.110) | 0.420 | 1.060(1.000-1.130) | 0.051 | 1.040(0.999-1.090) | 0.057 |  |
| Previous history |  |  |  |  |  |  |  |
| Diabetes | 1.120(0.759-1.660) | 0.560 | 1.270(0.924-1.740) | 0.142 | 1.230(0.984-1.530) | 0.069 |  |
| Hypertension | 1.550(1.050-2.290) | 0.029 | 0.949(0.705-1.280) | 0.728 | 1.070(0.870-1.320) | 0.514 |  |
| Dyslipidemia | 0.779(0.548-1.110) | 0.165 | 1.270(0.960-1.690) | 0.094 | 0.931(0.763-1.140) | 0.484 |  |
| Current smoker | 0.709(0.382-1.310) | 0.273 | 0.769(0.468-1.270) | 0.301 | 0.797(0.569-1.120) | 0.189 |  |
| Prior MI | 1.270(0.730-2.210) | 0.398 | 1.660(1.090-2.520) | 0.018 | 1.380(1.010-1.890) | 0.044 |  |
| Previous stroke | 2.220(1.470-3.350) | <0.001 | 1.010(0.645-1.570) | 0.980 | 1.250(0.936-1.660) | 0.131 |  |
| Previous PCI | 1.160(0.719-1.860) | 0.551 | 2.280(1.650-3.160) | <0.001 | 1.470(1.140-1.900) | 0.003 |  |
| COPD | 1.660(0.528-5.210) | 0.386 | - | - | 1.980(1.090-3.610) | 0.025 |  |
| Clinical presentation |  |  |  |  |  |  |  |
| Stable angina | Ref. |  | Ref. |  | Ref. |  |  |
| Unstable angina | 1.240(0.917-1.680) | 0.162 | 0.987(0.760-1.280) | 0.924 | 1.200(1.010-1.440) | 0.040 |  |
| NSTEMI | 1.030(0.710-1.490) | 0.881 | 0.859(0.633-1.170) | 0.328 | 0.993(0.802-1.230) | 0.952 |  |
| STEMI | 1.130(0.737-1.730) | 0.573 | 0.911(0.646-1.280) | 0.595 | 0.969(0.757-1.240) | 0.800 |  |
| eGFR, ml/min | 0.980(0.969-0.991) | <0.001 | 0.994(0.984-1.000) | 0.278 | 0.981(0.974-0.987) | <0.001 |  |
| LVEF | 1.010(0.982-1.030) | 0.567 | 0.988(0.970-1.010) | 0.214 | 0.984(0.971-0.996) | 0.010 |  |
| LVEDD | 1.020(0.979-1.050) | 0.404 | 1.020(0.994-1.060) | 0.120 | 1.030(1.010-1.050) | 0.006 |  |
| Baseline laboratory |  |  |  |  |  |  |  |
| WBC_group | 1.160(0.946-1.430) | 0.151 | 1.340(1.120-1.590) | 0.001 | 1.330(1.180-1.510) | <0.001 |  |
| WBC_Low | Ref. |  | Ref. |  | Ref. |  |  |
| WBC_Mid | 1.160(0.755-1.770) | 0.504 | 1.530(1.060-2.220) | 0.025 | 1.300(1.010-1.690) | 0.043 |  |
| WBC_High | 1.350(0.894-2.050) | 0.152 | 1.830(1.270-2.620) | 0.001 | 1.770(1.390-2.260) | <0.001 |  |
| Hemoglobin | 0.998(0.988-1.010) | 0.628 | 1.010(0.997-1.010) | 0.234 | 0.997(0.992-1.000) | 0.352 |  |
| Creatinine | 1.010(1.010-1.010) | <0.001 | 1.000(0.999-1.010) | 0.216 | 1.010(1.000-1.010) | <0.001 |  |
| Blood glucose | 1.020(0.984-1.070) | 0.236 | 1.020(0.983-1.050) | 0.321 | 1.030(1.010-1.060) | 0.003 |  |
| Total cholesterol (mg/dl) | 0.828(0.693-0.990) | 0.038 | 1.130(0.990-1.290) | 0.071 | 0.994(0.902-1.090) | 0.902 |  |
| TG | 0.858(0.711-1.040) | 0.111 | 1.050(0.944-1.160) | 0.392 | 0.984(0.904-1.070) | 0.702 |  |
| HDL | 0.714(0.308-1.650) | 0.431 | 0.807(0.404-1.610) | 0.542 | 0.871(0.539-1.410) | 0.571 |  |
| LDL | 0.804(0.636-1.020) | 0.068 | 1.160(0.974-1.380) | 0.096 | 1.010(0.886-1.140) | 0.924 |  |
| SYNTAX score | 1.020(1.000-1.050) | 0.021 | 1.020(1.000-1.040) | 0.018 | 1.030(1.010-1.040) | <0.001 |  |

HR: Hazard ratio; BMI: body mass index; MI: myocardial infarction; PCI: percutaneous coronary intervention; CI: confidence interval; COPD: chronic obstructive pulmonary disease; NSTEMI: non-ST-segment elevation myocardial infarction; STEMI: ST-segment elevation myocardial infarction; eGFR: estimated glomerular filtration rate; LVEF: left ventricular ejection fraction; LVEDD: left ventricular end diastolic diameter; TG: triglyceride; HDL: high density lipoprotein; LDL: low density lipoprotein; MACCEs: major adverse cardiovascular and cerebrovascular events..
